# Performance of three multiplex real-time PCR assays for simultaneous detection of 12 infectious pathogens in mice affected with respiratory and digestive diseases

**DOI:** 10.3389/fvets.2024.1421427

**Published:** 2024-08-20

**Authors:** Hye-young Wang, Jaeil Ahn, Jonghoon Lee, Sang Chul Kang, Hyunil Kim

**Affiliations:** ^1^Optipharm, Inc., Cheongju, Republic of Korea; ^2^Optipharm Animal Disease Diagnostic Center, Cheongju, Republic of Korea

**Keywords:** mouse, health monitoring, multiplex real-time PCR, diagnosis, sequence analysis

## Abstract

**Introduction:**

Research quality can be improved with reliable and reproducible experimental results when animal experiments are conducted using laboratory animals with guaranteed microbiological and genetic quality through health monitoring. Therefore, health monitoring requires the rapid and accurate diagnosis of infectious diseases in laboratory animals.

**Methods:**

This study presents a performance evaluation of a commercially available multiplex real-time PCR (mRT-PCR) assay for the rapid detection of 12 infectious pathogens (Set 1: Sendai virus [SeV, formally murine respirovirus], *Mycoplasma* spp., *Rodentibacter pneumotropicus*, and *Rodentibacter heylii*; Set 2: *Helicobacter* spp., Murine norovirus [MNV], Murine hepatitis virus [MHV], and *Salmonella* spp.; Set 3: *Staphylococcus aureus*, *Streptobacillus moniliformis*, *Corynebacterium kutscheri*, and *Pseudomonas aeruginosa*). To evaluate the efficacy of the mRT-PCR assay, 102 clinical samples encompassing fecal and cecal specimens were analyzed. The resulting data were then compared with the findings from sequence analysis for validation.

**Results:**

The assay’s detection limit ranged from 1 to 100 copies per reaction. Specificity testing involving various viruses and bacteria indicated no cross-reactivity between strains. Additionally, the assay exhibited good reproducibility, with mean coefficients of variation for inter- and intra assay variation below 3%. The overall positive rate was 52.9% (*n* = 54), with the mRT-PCR assay findings matching sequence analysis results (*κ* = 1). MHV (*n* = 29, 28.4%) was the most prevalent pathogen, followed by *Helicobacter* spp. (*n* = 28, 27.5%), *R. heylii* (*n* = 18, 17.6%), *Mycoplasma* spp. (*n* = 14, 13.7%), MNV (*n* = 12, 11.8%), *S. aureus* (*n* = 9, 8.8%), *P. aeruginosa* (*n* = 4, 3.9%), and *R. pneumotropicus* (*n* = 1, 0.9%).

**Discussion:**

This assay offers a rapid turnaround time of 100 min, including 30 min for DNA preparation and 70 min for target DNA/RNA amplification. It ensures accuracy, minimizing false positives or negatives, making it a convenient tool for the simultaneous detection of infectious diseases in many samples. Overall, the propose‑d assay holds promise for the effective detection of the most important pathogens in laboratory animal health monitoring.

## Introduction

Laboratory mice and rats are now widely used in animal experiments in various fields of research. To obtain reliable and reproducible results from animal experiments, laboratory animals must be maintained in a protected environment to avoid microbiological interference, and quality must be guaranteed through health monitoring (1). Therefore, microbiological quality control should be performed regularly to determine whether laboratory animals are free from various pathogens, including viruses, bacteria, fungi, and parasites (2).

Guidelines for the health monitoring of experimental animals were published by the Federation of European Laboratory Animal Science Associations (FELASA) (3). Until now, the health monitoring of laboratory animals has relied primarily on microscopic and culture-based methods (4). Serological test methods, such as enzyme-linked immunosorbent assay for primary screening or immunofluorescence assay (IFA) for confirmation, are mainly used to detect viruses or bacteria that are difficult to culture (5, 6). Due to the propensity for nonspecific reactions leading to false positives with these analysis methods, microbial quality control has been concurrently conducted alongside molecular diagnostic assays like polymerase chain reaction (PCR) or reverse transcription-polymerase chain reaction (RT-PCR) for cross-validation purposes (7–10). These assays are expensive, exhibit low sensitivity, require electrophoresis of the amplification products, and pose a contamination risk, potentially compromising result accuracy. Real-time (RT) PCR assay, the so-called quantitative PCR, has been a powerful analytical tool for detecting pathogenic bacteria or viruses since their development (11). Given its remarkable speed, heightened sensitivity, and precision, RT-PCR finds extensive use in pathogen diagnosis spanning animals, humans, and plants, and is additionally employed in laboratory animal studies (12–20).

Pathogens are commonly found in various systems, such as the respiratory, digestive, nervous, and other systems of laboratory animals. Sendai virus (SeV, formally murine respirovirus), *Mycoplasma* spp., murine hepatitis virus (MHV), Murine norovirus (MNV), and *Helicobacter* spp. are the primary pathogens found in the respiratory and digestive systems that pose marked interference with experimental results (2, 21, 22). In this study, we developed three sets of multiplex real-time PCR (mRT-PCR) assays that can simultaneously detect four different viral or bacterial pathogens causing respiratory (Opti SeV/MP/Rpn/Rhey Multi-qPCR; Sendai virus, *Mycoplasma* spp., *Rodentibacter pneumotropicus*, *R. heylii*), digestive (Opti Hel/MNV/MHV/Sal Multi-qPCR; *Helicobacter* spp., MNV, MHV, *Salmonella* spp.), and abscess or sepsis (Opti Sau/Smo/Cku/Pae Multi-qPCR; *S. aureus, S. moniliformis, C. kutscheri, P. aeruginosa*) diseases. We also confirmed their performance evaluations. The clinical performance of the mRT-PCR assay was evaluated using clinical samples requested for health monitoring of mice and rats, and the results were confirmed by sequence analysis.

## Methods

### Sample preparation

Seven viruses (SeV, MNV, MHV, Murine adenovirus, Ectromelia virus, Mouse minute virus, and Murine rotavirus) and thirty-five bacterial strains for specificity testing were provided by Xpressbio, the International Council for Laboratory Animal Science (ICLAS), and the American Type Culture Collection (ATCC). Provided by the Optipharm Animal Disease Diagnostic Center commissioned from 2020 to 2023, 102 fecal/cecal samples and health statuses were used to evaluate the diagnostic performance of the mRT-PCR assay. According to the manufacturer’s recommendation, DNA/RNA was extracted from 100 μL of phosphate-buffered saline (PBS)-pretreated samples using a commercial automated system (Miracle-AutoXT Automated Nucleic Acid Extraction System, Intronbio, Seongnam, Republic of Korea). To avoid cross-contamination, all samples were individually processed and stored at −20°C. The content and purity of the extracted DNA/RNA were analyzed by measuring the absorbance at 260 and 280 nm using a spectrophotometer (Infinite 200 NanoQuant; Tecan, Switzerland).

### Single RT-PCR assay

The effectiveness of the mRT-PCR assay was evaluated by comparing it with a single RT-PCR assay from a previously published reference. Table 1 contains the primers and probes used for the single RT-PCR. Each RT-PCR was performed according to the methods described in the paper.

**Table 1:** Sequences of primers and probes used in comparative experiments of mRT-PCR assay.

Species	Target	Primer	Sequence (5′-3′)	References
SeV	NP	F	CAGAGGAGCACAGTCTCAGTGTTC	12
		R	TCTCTGAGAGTGCTGCTTATCTGTGT	
		P	TGCATCATCAGTCACACTTGGGCCTAGTA	
MHV	M	F	GGAACTTCTCGTTGGGCATTATACT	19
		R	ACCACAAGATTATCATTTTCACAACATA	
		P	ACATGCTACGGCTCGTGTAACCGAACTGT	
MNV	VP1	F	CCGCAGGAACGCTCAGCAG	14
		R	GGYTGAATGGGGACGGCCTG	
		P	ATGAGTGATGGCGCA	
*Mycoplasma* spp.	16S rDNA	F	GCRAAGCTATAGARATATAGTGGAGG	13
		R	GTTGCGYTCGTTGCRGGAC	
		P	TGGTGCATGGTTGTC	
*Staphylococcus aureus*	nuc	F	GGCATATGTATGGCAATTGTTTC	16
		R	CGTATTGCCCTTTCGAAACATT	
		P	ATTACTTATAGGGATGGCTATC	
*Streptobacillus moniliformis*	16S rDNA	F	GGTTATCCAGTCTAAGAGGTAAGTTCT	28
		R	AGAATGCTTAACACATGCAAATCTATG	
		P	CACGTTACTCACCAGTCCACCATGTCTCTTATCT	
*Salmonella* spp.	invA	F	AGCGTACTGGAAAGGGAAAG	15
		R	ATACCGCCAATAAAGTTCACAAAG	
		P	CGTCACCTTTGATAAACTTCATCGCA	
*Pseudomonas aeruginosa*	phzA2	F	CAACTGGACCACGGAAAGC	17
		R	GTCTCGAAGATCCGCACGT	

SeV, Sendai virus; MHV, Murine hepatitis virus; MNV, Murine norovirus; NP, nucleocapsid protein; M, membrane; VP1, viral protein 1.

### mRT-PCR assay

**Figure 1:**
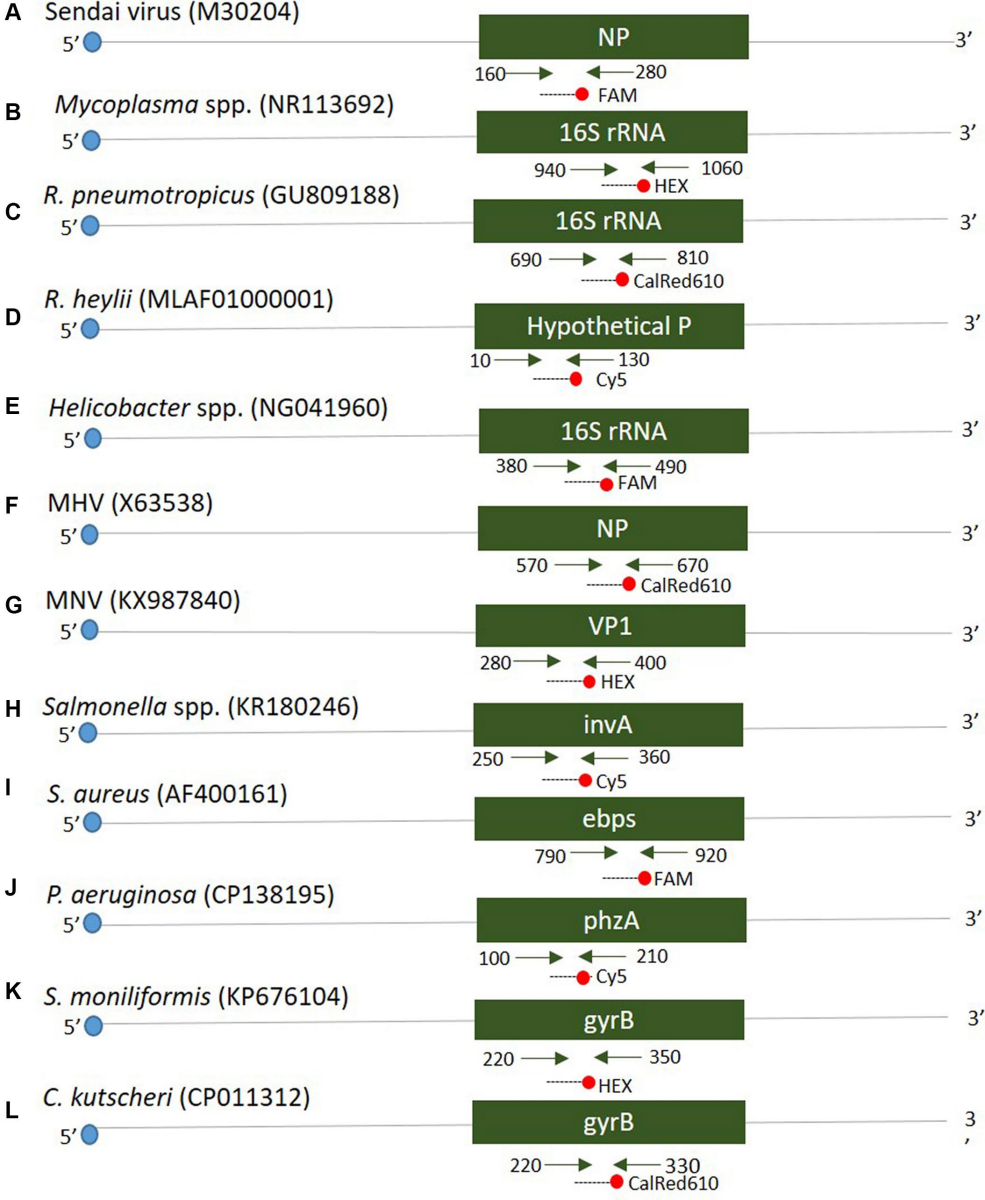
Schematic diagram distinguishing the positions of primers and probes of 12 target genes. A positive control was synthesized to verify the efficiency of the developed primers and probes. The mark in parentheses is the NCBI accession number.

Twelve oligonucleotide primers and probes corresponding to two strands were designed using Primer3Plus^1^ at the positions of each gene (see Figure 1). Primers were prepared as probes corresponding to the complementary strands and used exclusively thereafter. To verify the efficiency of the selected primers and probes, a positive control DNA sample was synthesized using Bioneer (Daejeon, Republic of Korea). The resulting product was mutagenized after subcloning with a pBHA vector. Clinical samples were screened for the presence of 12 diseases using Opti Multi-qPCR kits (Set 1: Opti SeV/MP/Rpn/Rhey; Set 2: Opti Hel/MNV/MHV/Sal; and Set 3: Opti Sau/Smo/Cku/Pae, Optipharm, Cheongju, Republic of Korea). These kits utilize a quantitative mRT-PCR-based assay and were processed using a CFX-96 RT-PCR system (Bio-Rad, Hercules, CA, USA) for both thermal cycling and fluorescence detection. RT-PCR amplification was performed in a total reaction volume of 20 μL containing 10 μL of 2× Thunderbird probe qPCR mix (Toyobo, Osaka, Japan), 2.5 μL of a mixture of 5 pmol each primer and 5 pmol TaqMan probe that were labeled with fluorophores (FAM/HEX-BHQ1, CalRed610/Cy5-BHQ2), and 3 μL template DNA/RNA. Positive (plasmid DNA) and negative controls comprising molecular grade (DNAse/RNAse-free) water (Ultra-pure water; Welgene, Gyeongsan, Republic of Korea) without template DNA/RNA were included in each assay. The assay was performed under the following conditions: For DNA, 95°C for 3 min, then 10 cycles of 3 s at 95°C and 30 s at 60°C, and then by 40 cycles of 3 s at 95°C and 30 s at 55°C; for RNA, 50°C for 2 min, 95°C for 2 min, then 10 cycles of 3 s at 95°C and 30 s at 60°C, and then by 40 cycles of 3 s at 95°C and 30 s at 55°C. Each sample was tested in duplicate by running the PCR cycle twice, and a positive result was obtained when the C_T_ value was below 35.

### Interfering reactions and reproducibility analysis

For interfering reactions, we used the following six substances by concentration: ethylene diamine tetraacetic acid (EDTA), sodium citrate (1, 10, 20, and 50 mM), heparin (250, 300, 375, and 500 IU) for anticoagulants, PBS (1X, 5X, 10X, and 20X) for tissue emulsion, EtOH and xylene (1, 5, 10, 20, and 50%), and blood (1, 5, and 10%). Assay repeatability and reproducibility were evaluated through 240 tests (10 days × 2 runs/day × 4 replicates × 3 lots). The coefficient of variation (CV) was calculated according to the mean C_T_ values/standard deviation (SD).

### Sequence analysis

To confirm the mRT-PCR assay results, PCR amplicons of positive clinical isolates were sequenced using an ABI 3,730 automated DNA sequencer (Applied Biosystems, Foster City, CA, USA) and the ABI Prism BigDye Terminator (Applied Biosystems) system (CosmoGenetech, Deajeon, Republic of Korea). The obtained sequence was compared with that of the National Center for Biotechnology Information GenBank database.

## Results

### Analytical sensitivity and specificity of the mRT-PCR assay

**Figure 2:**
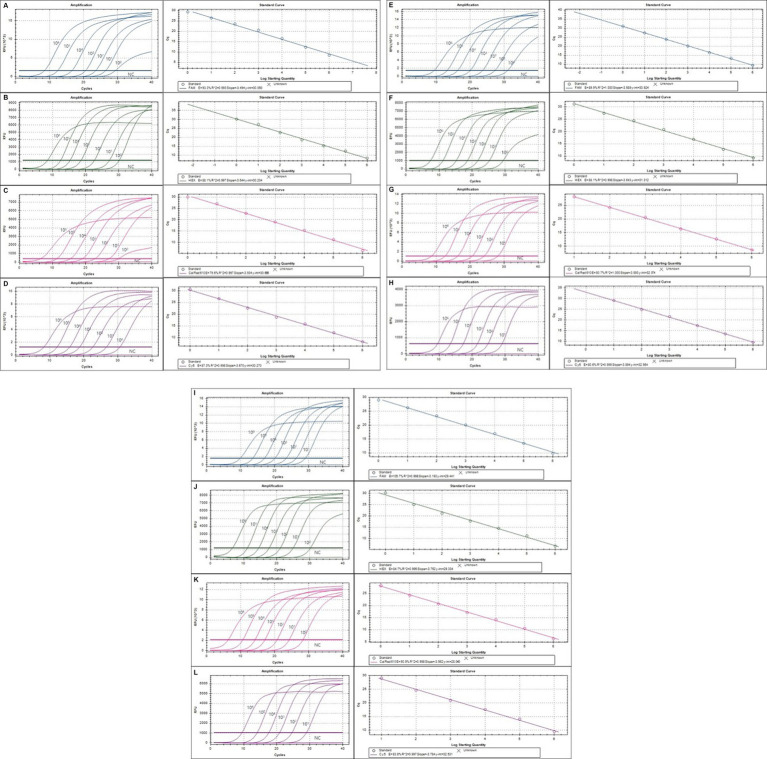
The detection limit of the multiplex real-time PCR (mRT-PCR) assay. The detection limit of the assay was evaluated using 10-fold serially diluted plasmid DNA. Each serially diluted control DNA, ranging from 10^6^ copies to 1 copy per reaction, was used to determine the detection limit of the RT-PCR assay. In the RT-PCR assay, the amplification curve of the specific probe for detecting SeV **(A)**, *Mycoplama* spp. **(B)**, *R. pneumotropicus* **(C)**, *R. heylii* **(D)**, *Helicobacter* spp. **(E)**, MNV **(F)**, MHV **(G)**, *Salmonella* spp. **(H)**, *S. aureus* **(I)**, *S. moniliformis* **(J)**, *C. kutscheri* **(K)**, and *P. aeruginosa* **(L)** is shown. The overall detection limit of this assay for each control DNA ranged from approximately 1 to 100 copy DNA per reaction. C_T_ was plotted against the input of the quantity of each DNA (repeated 10 times). The linearity was generated by plotting the log quantity of each DNA versus the corresponding C_T_ value, and the coefficient of determination of the linear regression was 0.993–1.0, with a slope ranging from −3.193 to −3.934. The fluorescence intensity is displayed on the *Y*-axis (*R*^2^ = reporter signal/passive reference signal). RFU, relative fluorescence unit; *R*^2^, fluorescence units.

The lower detection limit of the mRT-PCR assay for detecting three viruses (SeV, MNV, and MHV) and nine bacteria (*Mycoplasma* spp., *R. pneumotropicus*, *R. heylii*, *Helicobacter* spp., *Salmonella* spp., *S. aureus, S. moniliformis, C. kutscheri*, and *P. aeruginosa*) was measured with serial 10-fold dilutions (10^6^ copies to 1 copy) of the plasmid DNA stocks that contained each target gene sequence. Analytical sensitivity was estimated as the lowest number of gene copies yielding a positive result in all 10 replicates, and the corresponding C_T_ value was selected as the analysis cutoff. A standard curve was generated by plotting the log quantity of each plasmid DNA versus the corresponding C_T_ value, and the coefficient of determination (*R*^2^) for linear regression was 0.993–1.0, with a slope ranging from −3.193 to −3.934 (Figure 2). The detection limit of the mRT-PCR assay was detected at a concentration of approximately 1–100 copies per reaction. The mean C_T_ values of each DNA concentration ranged from 6.3 to 31.8, and the mean C_T_ (Standard deviation, SD) values were 6.45 ± 0.1 [95% confidence interval (CI), 6.4–6.5] to 31.4 ± 0.3 (95% CI, 31.1–31.8), and the CV was below 3% (Table 2).

**Table 2:** Analytical sensitivity of the mRT-PCR assay.

Con.	mRT-PCR Set1								mRT-PCR Set2								mRT-PCR Set3							
	Sen		MP		Rpn		Rhey		MHV		MNV		Hel		Sal		Sau		Smo		Cku		Pae	
	Ct ± SD	CV (%)	Ct ± SD	CV (%)	Ct ± SD	CV (%)	Ct ± SD	CV (%)	Ct ± SD	CV (%)	Ct ± SD	CV (%)	Ct ± SD	CV (%)	Ct ± SD	CV (%)	Ct ± SD	CV (%)	Ct ± SD	CV (%)	Ct ± SD	CV (%)	Ct ± SD	CV (%)
1 × 10^6^	8.42 ± 0.16	1.9	8.78 ± 0.15	1.8	6.45 ± 0.16	2.7	8.08 ± 0.2	2.4	8.74 ± 0.14	1.6	9.53 ± 0.25	2.6	9.44 ± 0.11	1.2	9.51 ± 0.12	1.3	9.8 ± 0.05	0.5	6.7 ± 0.18	2.8	7.58 ± 0.17	2.2	9.7 ± 0.24	2.5
1 × 10^5^	12.13 ± 0.17	1.4	12.66 ± 0.04	0.3	10.92 ± 0.2	1.8	11.95 ± 0.28	2.4	12.68 ± 0.03	0.3	13.72 ± 0.29	2.1	13.28 ± 0.32	2.4	13.31 ± 0.38	2.9	12.98 ± 0.29	2.2	11.1 ± 0.15	1.3	11.84 ± 0.06	0.5	14.32 ± 0.18	1.3
1 × 10^4^	15.59 ± 0.38	2.4	15.71 ± 0.38	2.4	14.66 ± 0.37	2.5	15.70 ± 0.39	2.5	16 ± 0.21	1.3	17.38 ± 0.25	1.5	16.7 ± 0.43	2.5	16.9 ± 0.43	2.6	16.72 ± 0.26	1.5	14.5 ± 0.13	0.9	15.47 ± 0.01	0.1	17.60 ± 0.08	0.4
1 × 10^3^	18.83 ± 0.51	2.7	18.98 ± 0.11	0.6	18.89 ± 0.13	0.7	19.14 ± 0.44	2.3	20.44 ± 0.18	0.9	20.93 ± 0.47	2.2	21.18 ± 0.4	1.9	21.09 ± 0.36	1.7	19.92 ± 0.34	1.7	17.5 ± 0.18	1.1	18.36 ± 0.09	0.5	20.7 ± 0.48	2.3
1 × 10^2^	22.49 ± 0.59	2.6	23.21 ± 0.08	0.3	22.59 ± 0.28	1.2	22.93 ± 0.44	1.9	24.04 ± 0.21	0.9	24.56 ± 0.16	0.6	24.58 ± 0.71	2.9	24.53 ± 0.59	2.4	23.25 ± 0.47	2	21 ± 0.21	1	22.02 ± 0.06	0.3	24.11 ± 0.33	1.4
1 × 10^1^	26.07 ± 0.62	2.4	26.66 ± 0.15	0.6	26.56 ± 0.5	1.9	26.36 ± 0.38	1.5	27.52 ± 0.45	1.6	27.9 ± 0.17	0.6	28.18 ± 0.81	2.9	28.27 ± 0.72	2.6	26.99 ± 0.6	2.2	24.4 ± 0.5	2.1	25.65 ± 0.21	0.8	28.89 ± 0.18	0.6
1 × 10^0^	30.13 ± 0.62	2.1	30.46 ± 0.26	0.9	30.4 ± 0.52	1.7	30.34 ± 0.19	0.6	–	–	31.48 ± 0.3	0.9	31.24 ± 0.83	2.7	–	–	30.1 ± 0.06	0.2	29.5 ± 0.53	1.8	29.2 ± 0.27	0.9	–	–
NC	–	–	–	–	–	–	–	–	–	–	–	–	–	–	–	–	–	–	–	–	–	–	–	–

Con, concentration; SeV, Sendai virus; MP, Mycoplasma spp.; Rpn, R. pneumotropicus; Rhey, R. heylii; MHV, Murine hepatitis virus; MNV, Murine norovirus; Hel, Helicobacter spp.; Sal, Salmonella spp.; Sau, S. aureus; Smo, S. moniliformis; Cku, C. kutscheri; Pae, P. aeruginosa; SD, standard deviation; CV, coefficient of variation.

To assess the potential cross-reactivity, analytical specificity analysis was performed using 50 strains, including 7 viruses, 35 bacteria, and 8 parasites. Three sets of mRT-PCR assays showed negative results in all strains, except for control DNA or RNA (Supplementary Figure S1). Hence, these three sets of 12 primers and probes did not react with any viral or bacterial strains (Table 3).

**Table 3:** Analytical specificity of the mRT-PCR assay with 42 strains.

No.	Species	Isolate	Sample type	mRT-PCR Set1				mRT-PCR Set2				mRT-PCR Set3			
				SeV (Ct)	MP (Ct)	Rpn (Ct)	Rhey (Ct)	Hel (Ct)	MNV (Ct)	MHV (Ct)	Sal (Ct)	Sau (Ct)	Smo (Ct)	Cku (Ct)	Pae (Ct)
1	*Sendai virus*	Xpressbio	Tissue	15.11	N/A	N/A	N/A	N/A	N/A	N/A	N/A	N/A	N/A	N/A	N/A
2	*Mycoplasma hominis*	ATCC 23,114	Tissue	N/A	18.46	N/A	N/A	N/A	N/A	N/A	N/A	N/A	N/A	N/A	N/A
3	*Rodentibacter pneumotropica*	ATCC 35,149	Tissue	N/A	N/A	12.67	N/A	N/A	N/A	N/A	N/A	N/A	N/A	N/A	N/A
4	*Rodentibacter heylii*	Field isolate	Tissue	N/A	N/A	N/A	17.58	N/A	N/A	N/A	N/A	N/A	N/A	N/A	N/A
5	*Helicobacter hepaticus*	Xpressbio	Tissue	N/A	N/A	N/A	N/A	25.28	N/A	N/A	N/A	N/A	N/A	N/A	N/A
6	*Murine norovirus*	ICLAS	Tissue	N/A	N/A	N/A	N/A	N/A	17.19	N/A	N/A	N/A	N/A	N/A	N/A
7	*Murine hepatitis virus*	ICLAS	Tissue	N/A	N/A	N/A	N/A	N/A	N/A	16.59	N/A	N/A	N/A	N/A	N/A
8	*Salmonella enteritidis*	ATCC 13,076	Tissue	N/A	N/A	N/A	N/A	N/A	N/A	N/A	25.14	N/A	N/A	N/A	N/A
9	*Salmonella typhi*	ATCC 19,430	Tissue	N/A	N/A	N/A	N/A	N/A	N/A	N/A	16.08	N/A	N/A	N/A	N/A
10	*Salmonella paratyphi*	ATCC BAA-1250	Tissue	N/A	N/A	N/A	N/A	N/A	N/A	N/A	25.46	N/A	N/A	N/A	N/A
11	*Salmonella newport*	ATCC 6,962	Tissue	N/A	N/A	N/A	N/A	N/A	N/A	N/A	26.2	N/A	N/A	N/A	N/A
12	*Salmonella typhimurium*	ATCC 14,028	Stool	N/A	N/A	N/A	N/A	N/A	N/A	N/A	12.62	N/A	N/A	N/A	N/A
13	*Staphylococcus aureus*	ATCC 25,923	Tissue	N/A	N/A	N/A	N/A	N/A	N/A	N/A	N/A	18.31	N/A	N/A	N/A
14	*Staphylococcus aureus*	ATCC 29,213	Stool	N/A	N/A	N/A	N/A	N/A	N/A	N/A	N/A	18.45	N/A	N/A	N/A
15	*Staphylococcus aureus*	ATCC 6,538	Sludge	N/A	N/A	N/A	N/A	N/A	N/A	N/A	N/A	13.6	N/A	N/A	N/A
16	*Streptobacillus moniliformis*	Xpressbio	Collagen	N/A	N/A	N/A	N/A	N/A	N/A	N/A	N/A	N/A	20.23	N/A	N/A
17	*Corynebacterium kutscheri*	Xpressbio	Collagen	N/A	N/A	N/A	N/A	N/A	N/A	N/A	N/A	N/A	N/A	20.84	N/A
18	*Pseudomonas aeruginosa*	ATCC 27,853	Tissue	N/A	N/A	N/A	N/A	N/A	N/A	N/A	N/A	N/A	N/A	N/A	9.65
19	*Bordetella bronchiseptica*	ATCC 4,617	Tissue	N/A	N/A	N/A	N/A	N/A	N/A	N/A	N/A	N/A	N/A	N/A	N/A
20	*Cilia-associated respiratory (CAR) bacillus*	Xpressbio	Tissue	N/A	N/A	N/A	N/A	N/A	N/A	N/A	N/A	N/A	N/A	N/A	N/A
21	*Citrobacter freundii*	ATCC 43,864	Tissue	N/A	N/A	N/A	N/A	N/A	N/A	N/A	N/A	N/A	N/A	N/A	N/A
22	*Citrobacter rodentium*	ATCC 51,116	Tissue	N/A	N/A	N/A	N/A	N/A	N/A	N/A	N/A	N/A	N/A	N/A	N/A
23	*Escherichia coli*	ATCC 25,922	Tissue	N/A	N/A	N/A	N/A	N/A	N/A	N/A	N/A	N/A	N/A	N/A	N/A
24	*Escherichia coli*	ATCC 35,150	Tissue	N/A	N/A	N/A	N/A	N/A	N/A	N/A	N/A	N/A	N/A	N/A	N/A
25	*Klebsiella oxytoca*	ATCC 700,324	Tissue	N/A	N/A	N/A	N/A	N/A	N/A	N/A	N/A	N/A	N/A	N/A	N/A
26	*Klebsiella pneumoniae*	ATCC 13,883	Tissue	N/A	N/A	N/A	N/A	N/A	N/A	N/A	N/A	N/A	N/A	N/A	N/A
27	*Streptococcus agalactiae*	ATCC 27,956	Tissue	N/A	N/A	N/A	N/A	N/A	N/A	N/A	N/A	N/A	N/A	N/A	N/A
28	*Streptococcus equi subsp. Zooepidemicus*	ATCC 43,079	Culture	N/A	N/A	N/A	N/A	N/A	N/A	N/A	N/A	N/A	N/A	N/A	N/A
29	*Streptococcus pneumoniae*	ATCC 49,619	Culture	N/A	N/A	N/A	N/A	N/A	N/A	N/A	N/A	N/A	N/A	N/A	N/A
30	*Streptococcus pyogenes*	ATCC 12,344	Culture	N/A	N/A	N/A	N/A	N/A	N/A	N/A	N/A	N/A	N/A	N/A	N/A
31	*Murine adenovirus*	Xpressbio	Culture	N/A	N/A	N/A	N/A	N/A	N/A	N/A	N/A	N/A	N/A	N/A	N/A
32	*Ectromelia virus*	ICLAS	Culture	N/A	N/A	N/A	N/A	N/A	N/A	N/A	N/A	N/A	N/A	N/A	N/A
33	*Mouse minute virus*	ICLAS	Culture	N/A	N/A	N/A	N/A	N/A	N/A	N/A	N/A	N/A	N/A	N/A	N/A
34	*Murine rotavirus*	ICLAS	Culture	N/A	N/A	N/A	N/A	N/A	N/A	N/A	N/A	N/A	N/A	N/A	N/A
35	*Pasteurella multocida*	ATCC 15,743	Culture	N/A	N/A	N/A	N/A	N/A	N/A	N/A	N/A	N/A	N/A	N/A	N/A
36	*Clostridium perfringens*	ATCC 13,124	Culture	N/A	N/A	N/A	N/A	N/A	N/A	N/A	N/A	N/A	N/A	N/A	N/A
37	*Campylobacter jejuni*	ATCC 29,428	Culture	N/A	N/A	N/A	N/A	N/A	N/A	N/A	N/A	N/A	N/A	N/A	N/A
38	*Campylobacter coli*	ATCC 33,559	Culture	N/A	N/A	N/A	N/A	N/A	N/A	N/A	N/A	N/A	N/A	N/A	N/A
39	*Listeria monocytogenes*	ATCC 35,152	Culture	N/A	N/A	N/A	N/A	N/A	N/A	N/A	N/A	N/A	N/A	N/A	N/A
40	*Shigella sonnei*	ATCC 25,931	Culture	N/A	N/A	N/A	N/A	N/A	N/A	N/A	N/A	N/A	N/A	N/A	N/A
41	*Shigella flexneri*	ATCC 12,022	Culture	N/A	N/A	N/A	N/A	N/A	N/A	N/A	N/A	N/A	N/A	N/A	N/A
42	*Yersinia enterocolitica*	ATCC 9,610	Culture	N/A	N/A	N/A	N/A	N/A	N/A	N/A	N/A	N/A	N/A	N/A	N/A
43	*Syphasia obvelata*	Field	Stool	N/A	N/A	N/A	N/A	N/A	N/A	N/A	N/A	N/A	N/A	N/A	N/A
44	*Syphasia muris*	Field	Stool	N/A	N/A	N/A	N/A	N/A	N/A	N/A	N/A	N/A	N/A	N/A	N/A
45	*Aspiculuris tetraptera*	Field	Stool	N/A	N/A	N/A	N/A	N/A	N/A	N/A	N/A	N/A	N/A	N/A	N/A
46	*Trichomonas muris*	Field	Stool	N/A	N/A	N/A	N/A	N/A	N/A	N/A	N/A	N/A	N/A	N/A	N/A
47	*Tritrichomonas muris*	Field	Stool	N/A	N/A	N/A	N/A	N/A	N/A	N/A	N/A	N/A	N/A	N/A	N/A
48	*Entamoeba muris*	Field	Stool	N/A	N/A	N/A	N/A	N/A	N/A	N/A	N/A	N/A	N/A	N/A	N/A
49	*Hexamita kirbyi*	Field	Stool	N/A	N/A	N/A	N/A	N/A	N/A	N/A	N/A	N/A	N/A	N/A	N/A
50	*Chilomastix mesnili*	Field	Stool	N/A	N/A	N/A	N/A	N/A	N/A	N/A	N/A	N/A	N/A	N/A	N/A
51	PC	–	–	18.41	19.57	18.07	19.65	25.57	24.95	19.33	23.79	18.57	17.05	17.82	16.36
52	NC	–	–	N/A	N/A	N/A	N/A	N/A	N/A	N/A	N/A	N/A	N/A	N/A	N/A

ATCC, American type culture collection; ICLAS, International Council for Laboratory Animal Science.

### Results of interfering reactions and reproducibility analysis using the mRT-PCR assay

We tested six substances at different concentrations to check for potential interference with the sample preparation, extraction, or qPCR progress. No interference was observed below these values: 30 mM EDTA, 40–50 mM sodium citrate, 375 IU heparin, 4-5X PBS, 50% EtOH, and 5% blood (Table 4). For repeatability and reproducibility, the measured number for the 3 concentrations (10^5^, 10^3^, and 10^1^) of each positive control DNA was 240 (10 days × 2 runs/day × 4 replicates × 3 lots). The CV for intra- and inter assay variability ranged from 0.1 to 0.5% and 0.1 to 1%, respectively (see Table 5), which were all below 3%. These experimental results suggest that the three sets of developed mRT-PCR assays may have stable results.

**Table 4:** Results of interfering reactions.

Substances	SeV/MP/Rpn/Rhey		Hel/MNV/MHV/Sal		Sau/Smo/Cku/Pae	
	Max	CV (%)	Max	CV (%)	Max	CV (%)
EDTA (mM)	30	2.7	30	1.3	30	1.8
Sodium citrate (mM)	50	3.5	40	4.7	50	2.2
Heparin (IU)	375	4.3	375	3.2	375	3.2
PBS (X)	4	2.3	4.5	2.9	5	1.6
EtOH (%)	50	1.8	50	1.5	50	2.2
Whole blood (%)	5	2.7	5	1.2	5	3.7

EDTA, ethylene diamine tetraacetic acid; PBS, phosphate-buffered saline; EtOH, ethanol; Max, maximum; CV, coefficient of variation.

**Table 5:** Results of intra- and inter assay for repeatability and reproducibility analysis.

mRT-PCR assay	Copies/μL	*N*	Total			Intra assay			Inter assay					
						Within-run			Between-run			Between-day		
			Mean Ct	SD	CV (%)	Mean Ct	SD	CV (%)	Mean Ct	SD	CV (%)	Mean Ct	SD	CV (%)
Set 1 (SeV/MP/Rpn/Rhey)	10^5^	240	7.3	0.0	0.2	7.3	0.0	0.3	7.3	0.0	0.4	7.3	0.1	0.7
	10^3^	240	14.9	0.1	0.4	14.8	0.1	0.5	14.9	0.0	0.2	14.9	0.1	1
	10^1^	240	23.9	0.1	0.2	23.9	0.1	0.3	23.9	0.1	0.2	23.9	0.1	0.6
Set 2 (Hel/MNV/MHV/Sal)	10^5^	240	9.1	0.0	0.3	9.1	0.0	0.3	9.1	0.0	0.4	9.1	0.1	0.9
	10^3^	240	16.6	0.1	0.3	16.6	0.0	0.2	16.6	0.0	0.3	16.6	0.1	0.7
	10^1^	240	25.0	0.1	0.3	25.1	0.0	0.1	24.9	0.1	0.4	25.0	0.2	0.7
Set 3 (Sau/Smo/Cku/Pae)	10^5^	240	10.5	0.0	0.3	10.6	0.0	0.3	10.5	0.1	0.7	10.5	0.1	0.8
	10^3^	240	17.6	0.1	0.3	17.5	0.1	0.3	17.6	0.1	0.6	17.6	0.1	0.8
	10^1^	240	25.6	0.0	0.1	25.6	0.0	0.2	25.5	0.1	0.3	25.6	0.1	0.4

*N*, test number of inter- and intraassay; SD, standard deviation; CV, coefficients of variation; Set 1, SeV/Mycobacteria spp./R. pneumotropicus/R. heylii; Set 2, Helicobacter spp./MNV/MHV/Salmonella spp.; Set 3, S. aureus/S. moniliformis/C. kutscheri/P. aeruginosa.

### Result comparison between single RT-PCR and mRT-PCR assays

**Figure 3:**
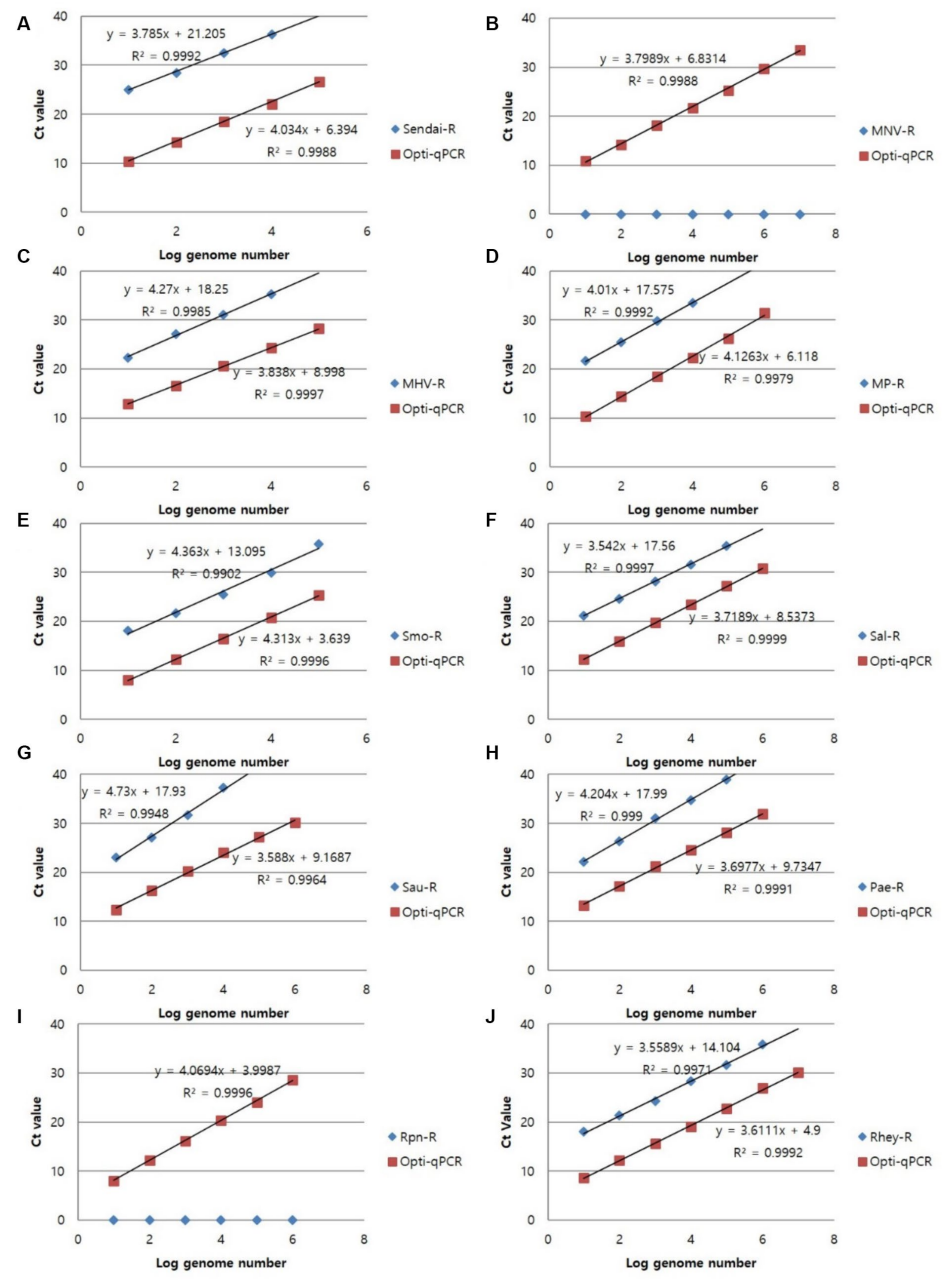
Representative results of the single RT-PCR and mRT-PCR assays according to the concentration of positive samples. Representative results of the two assays with positive samples at concentrations of 1 ng, 100 pg., 10 pg., 1 pg., 100 fg, and 10 fg. **(A)** SeV; **(B)** MNV; **(C)** MHV; **(D)** *Mycoplasma* spp.; **(E)** *S. moniliformis*; **(F)** *Salmonella* spp.; **(G)** *S. aureus*; **(H)** *P. aeruginosa*; **(I)** *R. pneumotropicus*; **(J)** *R. heylii*.

To confirm the analytical performance of the three sets of mRT-PCR assays, the concentration of each positive sample was compared with that of the single RT-PCR assay across 10 previous studies (SeV, MNV, MHV, *Mycoplasma* spp., *S. moniliformis*, *Salmonella* spp., *S. aureus*, *P. aeruginosa, R. pneumotropicus*, and *R. heylii*) (Table 1). The mRT-PCR assay demonstrated higher sensitivity than the single RT-PCR assay, except for MNV, *S. moniliformis*, and *R. pneumotropicus*. The single qPCR did not detect MNV and *R. pneumotropicus*, and *S. moniliformis* appeared similarly (Figure 3). Because research on *Helicobacter* spp. and *C. kutscheri* by single RT-PCR remains unavailable, comparative experiments could not be performed in this study.

### Detection of target genes using an mRT-PCR assay in clinical samples

In a pilot study, we tested the detection of three sets of mRT-PCR assays using 102 clinical samples, including feces and ceca. Among the 102 clinical samples, 54 (52.9%) samples were positive for one or more than seven target genes, whereas 48 (47.1%) samples were negative for all 12 target genes as detected by mRT-PCR. Of the 54 positive samples, 29 (28.4%) were MHV, 28 (27.5%) were *Helicobacter* spp., 18 (17.6%) were *R. heylii*, 14 (13.7%) were *Mycoplasma* spp., 12 (11.8%) were MNV, 9 (8.8%) were *S. aureus*, 4 (3.9%) were *P. aeruginosa*, and 1 (0.9%) were *R. pneumotropicus* (Table 6). The C_T_ values of positive samples ranged from 16.2 to 30.6 (mean 24.8, SD ± 2.8) in MHV, 14.2 to 33.4 (mean 21.4, SD ± 4.8) in *Helicobacter* spp., 21.5 to 28.7 (mean 24.6, SD ± 2.1) in *R. heylii*, 13.7 to 28.4 (mean 21.5, SD ± 4.9) in *Mycoplasma* spp., 12.9 to 27.8 (mean 19.3, SD ± 4.7) in MNV, 12.4 to 28.8 (mean 24.1, SD ± 5.1) in *S. aureus*, 5.2 to 27.7 (mean 18.8, SD ± 11.8) in *P. aeruginosa*, and 9.15 in *R. pneumotropicus*, respectively. To validate the mRT-PCR-derived results, a sequence analysis was performed using the same clinical samples. All 54 samples detected as 8-positive target genes by the mRT-PCR assay were consistent with the sequencing results (Table 6). Further investigation of single or multiple infections of the eight target genes identified as positive revealed that 18 (17.6%) had a single infection, and 36 (35.3%) were positive for two or more multiple infections (Table 7).

**Table 6:** Detection results of 12 target genes in 102 clinical samples using mRT-PCR assay.

No.	Target	Total no. (%) of samples	Ranged CT value (mean ± SD)	Sequence analysis, no. (%)/Consistent results	Sensitivity, % (*n*) (95% CI)	Specificity, % (*n*) (95% CI)	PPV, % (*n*) (95% CI)	NPV, % (*n*) (95% CI)	*κ* coefficient (95% CI)
1	MHV	29 (28.4)	16.2–30.6 (24.8 ± 2.8)	29 (100)	100 (29/29) (0.915–1.000)	100 (73/73) (0.964–1.000)	100 (29/29) (0.915–1.000)	100 (73/73) (0.964–1.000)	1 (0.937–1.000)
2	Hel	28 (27.5)	14.2–33.4 (21.4 ± 4.8)	28 (100)	100 (28/28) (0.912–1.000)	100 (74/74) (0.964–1.000)	100 (28/28) (0.912–1.000)	100 (74/74) (0.964–1.000)	1 (0.938–1.000)
3	Rhey	18 (17.6)	21.5–28.7 (24.6 ± 2.1)	18 (100)	100 (18/18) (0.869–1.000)	100 (84/84) (0.968–1.000)	100 (18/18) (0.869–1.000)	100 (84/84) (0.968–1.000)	1 (0.945–1.000)
4	MP	14 (13.7)	13.7–28.4 (21.5 ± 4.9)	14 (100)	100 (14/14) (0.838–1.000)	100 (88/88) (0.970–1.000)	100 (14/14) (0.838–1.000)	100 (88/88) (0.970–1.000)	1 (0.947–1.000)
5	MNV	12 (11.8)	12.9–27.8 (19.3 ± 4.7)	12 (100)	100 (12/12) (0.816–1.000)	100 (90/90) (0.970–1.000)	100 (12/12) (0.816–1.000)	100 (90/90) (0.970–1.000)	1 (0.949–1.000)
6	Sau	9 (8.8)	12.4–28.8 (24.1 ± 5.1)	9 (100)	100 (9/9) 0.768–1.000	100 (93/93) (0.971–1.000)	100 (9/9) 0.768–1.000	100 (93/93) (0.971–1.000)	1 (0.950–1.000)
7	Pae	4 (3.9)	5.2–27.7 (18.8 ± 11.8)	4 (100)	100 (4/4) (0.596–1.000)	100 (98/98) (0.973–1.000)	100 (4/4) (0.596–1.000)	100 (98/98) (0.973–1.000)	1 (0.953–1.000)
8	Rpn	1 (0.9)	9.15	1 (100)	100 (1/1) (0.269–1.000)	100 (101/101) 0.974–1.000	100 (1/1) (0.269–1.000)	100 (101/101) 0.974–1.000	1 (0.954–1.000)
9	SeV	–	–	–	–	–	–	–	–
10	Sal	–	–	–	–	–	–	–	–
11	Smo	–	–	–	–	–	–	–	–
12	Cku	–	–	–	–	–	–	–	–

MHV, Murine hepatitis virus; Hel, Helicobacter spp.; Rhey, R. heylii; MP, Mycoplasma spp.; MNV, Murine norovirus; Sau, S. aureus; Pae, P. aeruginosa; Rpn, R. pneumotropicus; SeV, Sendai virus; Sal, Salmonella spp.; Smo, S. moniliformis; Cku, C. kutscheri; PPV, positive predictive value; NPV, negative predictive value; 95% CI, 95% confidence interval.

**Table 7:** Results of single and multiple infections in 102 clinical samples.

mRT-PCR assay	No (%) of samples
Positive	54 (52.9)
Single infection	18 (17.6)
MHV	6 (5.9)
*P. aeruginosa*	4 (3.9)
*S. aureus*	2 (1.9)
*Helicobacter* spp.	2 (1.9)
*R. heylii*	2 (1.9)
MNV	1 (0.9)
*R. pneumotropicus*	1 (0.9)
Multiple infection	36 (35.3)
*R. heylii* + *Helicobacter* spp.	6 (5.9)
MHV + *S. aureus*	6 (5.9)
MHV + *Helicobacter* spp.	4 (3.9)
MNV + *Helicobacter* spp.	2 (1.9)
MNV + *P. aeruginosa*	1 (0.9)
*Mycoplasma* spp. + *Helicobacter* spp. + MHV	2 (1.9)
*Mycoplasma* spp. + *Helicobacter* spp. + MNV	2 (1.9)
MHV + MNV + *R. heylii*	2 (1.9)
*Mycoplasma* spp. + *Helicobacter* spp. + *R. heylii*	1 (0.9)
*Mycoplasma* spp. + MHV + *S. aureus*	1 (0.9)
*Helicobacter* spp. + MHV + *R. heylii*	1 (0.9)
*Mycoplasma* spp. + *Helicobacter* spp. + MHV + *R. heylii*	4 (3.9)
*Mycoplasma* spp. + *Helicobacter* spp. + MHV + MNV	3 (2.9)
*Mycoplasma* spp. + *Helicobacter* spp. + MNV + *R. heylii*	1 (0.9)
Negative	48 (47.1)
Total	102 (100)

## Discussion

Health monitoring of laboratory animals is a fundamental aspect of healthcare, serving as a crucial prerequisite for both animal welfare and scientifically rigorous research (23). Obtaining reliable and reproducible experimental results requires using animals with guaranteed microbiological and genetic quality (24). Therefore, health monitoring necessitates accurate and rapid diagnosis of infectious diseases in laboratory animals.

This study aimed to assess the analytical performance and clinical effectiveness of three newly developed mRT-PCR assays, leveraging both conventional PCR and multiplex PCR techniques to enable rapid and precise simultaneous detection. The development of the mRT-PCR assay for 12 pathogens affecting respiratory (Set 1, SeV/*Mycoplasma* spp./*R. pneumotropicus/R. heylii*) and digestive systems (Set 2, *Helicobacter* spp./MNV/MHV/*Salmonella* spp.) and abscess/sepsis (Set 3, *S. aureus/S. moniliformis/C. kutscheri/P. aeruginosa*) was prompted by recommendations outlined in the Korea Laboratory Animal Microbiological Standards and Monitoring (1108-01, 2021) guidelines. These guidelines recommend regular testing for these pathogens every three months, particularly for SPF-grade laboratory rats, mice, guinea pigs, and rabbits. Although conventional PCR or single RT-PCR has been used to isolate these diseases, mRT-PCR assays for the simultaneous differentiation of these viruses or bacteria remain lacking. An mRT-PCR assay enables the detection of various multiple genes in the same reaction tube by using 4–5 fluorescent dyes in RT-PCR equipment. It has the same advantages as conventional RT-PCR, including ease of use, rapid turnaround time of 70 min, accuracy, reproducibility, low risk of cross-contamination due to sequential reactions in the same tube, and high-throughput capacity to enable quick screening of multiple samples (25, 26). This assay is also a rapid method with a total turnaround time of approximately 100 min, including 30 min for DNA preparation and 70 min for target DNA amplification. In particular, RNA samples are also processed in one step, including cDNA synthesis, and the overall amplification time is similar to that of DNA samples. Considering the cost and availability in commercial laboratories, the cost of a single RT-PCR to diagnose one gene is usually $10 per test. If you would like to diagnose four genes with a single RT-PCR, you should need $40, whereas the mRT-PCR assay that diagnoses four genes simultaneously costs $15 per test. Therefore, we believe that the cost can be reduced by about 62.5%, making it highly usable in commercial laboratories.

The analytical performance evaluations of sensitivity, specificity, interference response, and reproducibility conducted in this study are the performance evaluation data required for product approval in Korea. The results of the analytical performance evaluation of the developed mRT-PCR are summarized as follows: (i) the detection limit of this assay was measured from 1 to 100 copies, suggesting that pathogens can be detected even at low amounts; (ii) in the specificity test using various viruses and bacteria, no cross-reactivity was observed between strains. The presence of other samples within the 12 infected strains did not affect the assay performance. (iii) Regarding interference reactions, some substances differed from the results of previous studies (26), and the variations in concentration causing interference were sample-specific. (iv) This assay showed high reproducibility with mean CVs of inter- and intra assay variation of below 3%. Furthermore, this assay demonstrated consistent results over a 12 month period, as verified by an external accelerated aging test conducted by KTC (Gunpo, Republic of Korea) (data not shown). A comparison analysis of the results of single RT-PCR assays used in previous studies validated the high sensitivity of the mRT-PCR assay.

In this study, the mRT-PCR-derived results were consistent with sequence analysis findings as per the clinical samples used, indicating a high level of agreement (*κ* = 1). Using sequence analysis as the gold standard, the sensitivity, specificity, and positive and negative predictive values of the results by mRT-PCR assay were 100% (*n* = 54, 95% CI 0.952–1.000, *p* < 0.001), 100% (*n* = 48, 95% CI 0.946–1.000, *p* < 0.001), 100% (95% CI 0.952–1.000, *p* < 0.001), and 100% (95% CI 0.946–1.000, *p* < 0.001), respectively. In a previous study, MNV (25.9%) and MHV (3.9%) for viruses and *Helicobacter* spp. (21%), *Pasteurella pneumotropica* (18.3%), and *S. aureus* (9.1%) for bacteria were the most prevalent pathogens identified in mice. Our results showed similar results, with MHV (28.4%) and MNV (11.8%) for viral, *Helicobacter* spp. (27.5%), *Rodentibacter* spp. (18.5%, *R. heylii* 17.6% and *R. pneumotropicus* 0.9%), *Mycoplama* spp. (13.7%), and *S. aureus* (8.8%) (27, 28). *P. pneumotropica* was recently reclassified to a new genus *Rodentibacter*, with *R. pneumotropicus* and *R. heylii* as the most commonly found species in laboratory mouse colonies (20). Until now, differentiation between *R. pneumotropicus* and *R. heylii* by culture or PCR has proven challenging, but we believe that the mRT-PCR assay will enable testing to distinguish between the two species. Multiple infections (*n* = 36, 35.3%) with two or more pathogens exhibited a higher positivity rate than single infections (*n* = 18, 17.6%). Confirming the ratio was difficult owing to the lack of simultaneous diagnosis of multiple pathogens, unlike our results. However, because the detection rate of *Mycoplama* spp. was high (13.7%) compared to the previous study’s 0.47%, the contaminated environment of experimental animals is assumed to have been affected.

This study has certain limitations. (i) Because our clinical results were tested on random samples requested for health monitoring, 4 (SeV, *Salmonella* spp., *S. moniliformis*, and *C. kutscheri*) out of 12 pathogens were not detected. Hence, future studies will require additional tests with larger sample sizes to further validate these findings. (ii) Endo and ecto parasites are a significant threat to the biosecurity of rodent research colonies. In pilot study, we tested the detection of three sets of mRT-PCR assays using 32 parasite samples, including *Syphacia* spp. and *Aspiculuris tetreptera*, known as pinworms, confirmed by microscopy and sequencing. As a result, these three sets of mRT-PCR assays did not react in all 32 parasite samples (data not shown). Likewise, not all samples related to endo and ecto parasites were tested, so additional testing is required.

## Conclusion

The developed mRT-PCR assay consistently showed high agreement and specificity with the sequence analysis. This assay offers rapidity and accuracy, effectively minimizing the risk of false positives or false negatives. It is a convenient tool for simultaneously detecting the presence of infectious diseases in numerous samples. Therefore, the use of the newly developed mRT-PCR assay will prove beneficial in detecting the most crucial diseases during the health monitoring of laboratory animals. We believe that this assay can serve as a sensitive and specific tool to complement or replace traditional methods because it can reduce the labor and time required for diagnosis in the field of laboratory animals.

## Data availability statement

The original contributions presented in the study are included in the article/Supplementary material, further inquiries can be directed to the corresponding authors.

## Ethics statement

The animal studies were approved by written informed consent was obtained from the owners for the participation of their animals in this study. The studies were conducted in accordance with the local legislation and institutional requirements. Written informed consent was obtained from the owners for the participation of their animals in this study.

## Author contributions

H-yW: Conceptualization, Data curation, Formal analysis, Investigation, Methodology, Validation, Writing – original draft, Writing – review & editing. JA: Writing – review & editing. JL: Writing – original draft, Methodology. SK: Writing – original draft, Investigation. HK: Writing – original draft, Conceptualization, Supervision.

## Footnotes

^1^ http://www.bioinformatics.nl/cgi-bin/primer3plus/primer3plus.cgi
