# Supplementary material for: Performance of three multiplex real-time PCR assays for simultaneous detection of 12 infectious pathogens in mice affected with respiratory and digestive diseases
Source: Front Vet Sci. 2024 Aug 20;11:1421427. doi: 10.3389/fvets.2024.1421427 (PMC11370653; doi:10.3389/fvets.2024.1421427)
Supplement: Supplementary file 1 [file Data_Sheet_1.docx]

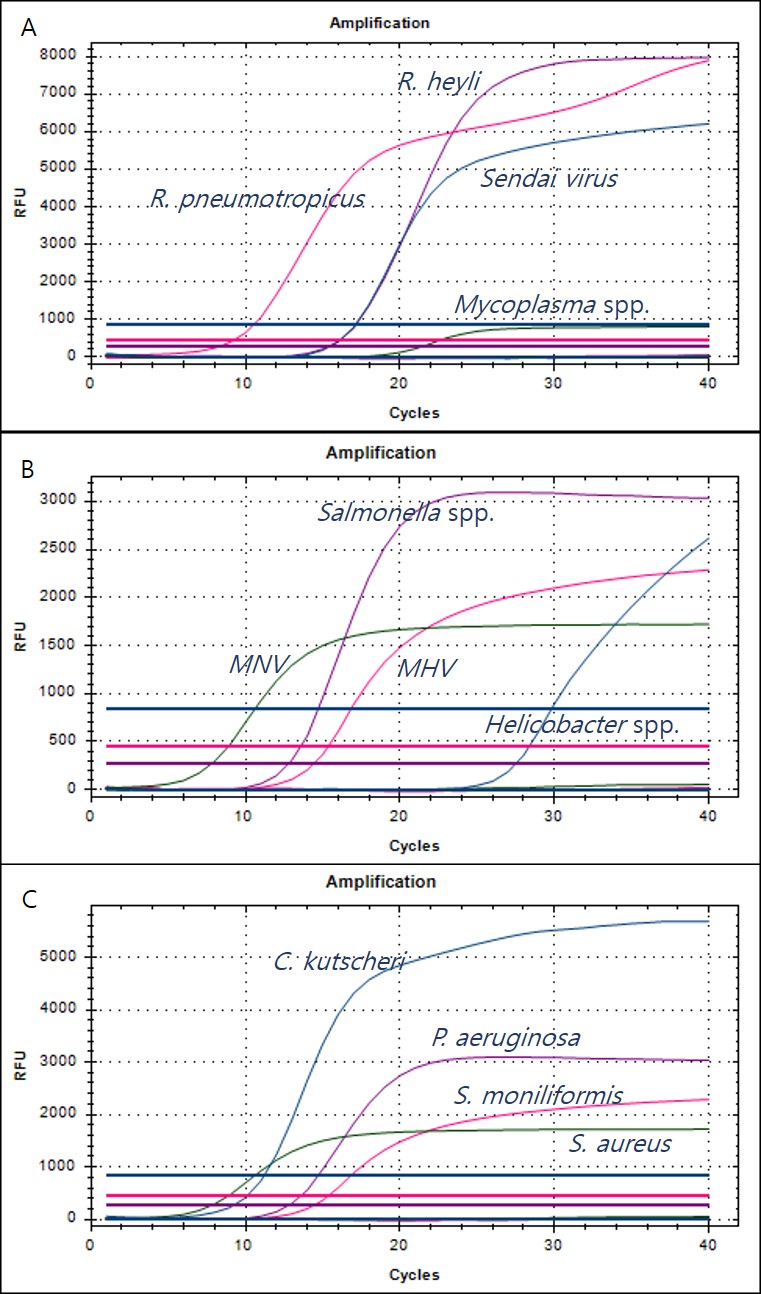


**(Supplementary) FIGURE 1** Example of analytical sensitivity of the mRT-PCR assay. A, Multi-Set 1: SeV/*Mycoplasma* spp./*R. puemotropicus/R. heylii*; B, Set 2: *Helicobacter* spp./MNV/MHV/*Salmonella* spp.; C, Set 3: *S. aureus/S. moniliformis/C. kutscheri/P. aeruginosa*. The straight line at the bottom of each figure represents the total number of cycles of mRT-PCR assay.
